# Effect of simulated tillage on microbial autotrophic CO_2_ fixation in paddy and upland soils

**DOI:** 10.1038/srep19784

**Published:** 2016-01-22

**Authors:** Tida Ge, Xiaohong Wu, Qiong Liu, Zhenke Zhu, Hongzhao Yuan, Wei Wang, A. S. Whiteley, Jinshui Wu

**Affiliations:** 1 Changsha Research Station for Agricultural and Environmental Monitoring & Key Laboratory of Agro-ecological Processes in Subtropical Region, Institute of Subtropical Agriculture, Chinese Academy of Sciences, Hunan, 410125, China; 2 State Key Laboratory of Soil and Sustainable Agriculture, Institute of Soil Science, Chinese Academy of Sciences, Jiangshu, 210008, China; 3 Faculty of Life Science and Technology, Central South University of Forestry and Technology, Changsha, 410004, China; 4 ISA-CAS and UWA Joint Laboratory for Soil Systems Biology, Hunan, 410125, China; 5 School of Earth and Environment, The University of Western Australia, Crawley, WA 6009, Australia

## Abstract

Tillage is a common agricultural practice affecting soil structure and biogeochemistry. To evaluate how tillage affects soil microbial CO_2_ fixation, we incubated and continuously labelled samples from two paddy soils and two upland soils subjected to simulated conventional tillage (CT) and no-tillage (NT) treatments. Results showed that CO_2_ fixation (^14^C-SOC) in CT soils was significantly higher than in NT soils. We also observed a significant, soil type- and depth-dependent effect of tillage on the incorporation rates of labelled C to the labile carbon pool. Concentrations of labelled C in the carbon pool significantly decreased with soil depth, irrespective of tillage. Additionally, quantitative PCR assays revealed that for most soils, total bacteria and *cbbL*-carrying bacteria were less abundant in CT versus NT treatments, and tended to decrease in abundance with increasing depth. However, specific CO_2_ fixation activity was significantly higher in CT than in NT soils, suggesting that the abundance of *cbbL*-containing bacteria may not always reflect their functional activity. This study highlights the positive effect of tillage on soil microbial CO_2_ fixation, and the results can be readily applied to the development of sustainable agricultural management.

Autotrophic bacteria in terrestrial ecosystems can partially compensate for increasing atmospheric CO_2_ concentration, predicted to reach 450–600 ppm by 2050^1^. These bacteria have the capacity to fix CO_2_ and are widely distributed in agricultural soils^2^,^3^,^4^. Among the six pathways developed by microbial autotrophs for CO_2_ fixation^5^, autotrophic bacteria predominantly use the Calvin–Benson–Bassham cycle. This pathway depends on the activity of ribulose-1,5-bisphosphate carboxylase/oxygenase (RubisCO), encoded by the *cbbL* gene^6^. Recently, environmental studies based on *cbbL* gene detection have shown that autotrophic bacteria are sensitive to agricultural management practices such as fertiliser treatment, land use alteration, and cropping systems^2^,^7^,^8^,^9^. Changes in soil physical, chemical, and biological properties caused by different management practices are reported to affect the abundance, diversity, and activity of CO_2_-fixing autotrophic bacteria and their associated fixation rates^2^,^4^,^7^,^8^,^9^.

Conventional tillage (CT), including ploughing and disking, has been the dominant agricultural practice for the past century of crop production^10^,^11^. Such management practices are excellent for loosening soil, which improves surface soil compaction, thus repressing annual weeds and benefiting precise seeding^12^,^13^. However, the intensive mechanical disturbance of soil structures introduced by CT practices is accompanied by surface soil erosion, a reduction in soil aggregate stability, and the acceleration of soil organic matter decomposition^14^,^15^. On-going changes in CT soil properties (e.g. porosity, bulk density, and organic carbon concentration) will affect water, gas, and nutrient diffusion, potentially triggering changes in soil bacterial communities^16^,^17^. Many studies have shown that CT practices negatively affect soil bacterial populations, often resulting in a decrease in community abundance, diversity, and activity when compared to no-tillage (NT) management^18^,^19^,^20^,^21^. However, this phenomenon is not observed in all reports, indicating that the underlying mechanisms driving the changes within CT soil bacterial communities are likely linked to a wide range of factors, including soil texture and depth of tillage^20^,^21^. Despite intensive studies concerning the impact of different tillage practices on soil bacterial communities, we still have limited knowledge about the ecological functions of specific microbial communities under various tillage managements. Our current understanding is that soil autotrophic bacteria do play a central role—modulated by tillage practices—in mitigating atmospheric CO_2_ emission^4^,^22^,^23^, but little data exist to clarify that role. Now, however, advances in molecular microbial ecology allow us to investigate the function of soil autotrophic bacteria by quantifying *cbbL* gene abundances^24^ and their associated CO_2_ fixation rates under different tillage managements. This method fills a major knowledge gap in clarifying the effects of tillage upon important global C sequestration processes.

The objective of this study was to evaluate the effect of different tillage practices on soil autotrophic bacterial populations and their CO_2_ assimilation rates at varying soil depths. Soils were sieved to experimentally generate conventional tillage treatment soils (CT), and intact soil cores without sieving were collected as the corresponding no-till treatment soils (NT). Using continuous labelling with ^14^CO_2_, we quantified the carbon fixed by soil autotrophs (^14^C-SOC), the distribution of newly assimilated carbon in the soil microbial biomass carbon pool (^14^C-MBC), and the dissolved organic carbon pool (^14^C-DOC) at different depths (0–1, 1–5, and 5–17 cm) of both CT and NT soils. Real-time quantitative PCR analysis was also conducted to assess how the abundance of autotrophic bacteria changed in response to tillage. We hypothesised that the mechanical disturbance from CT practices would decrease soil autotrophic bacterial abundance, leading to lower rates of CO_2_ fixation when compared with NT soils.

## Results

### Soil autotrophic bacteria CO_2_ fixation rate

**Figure 1:**
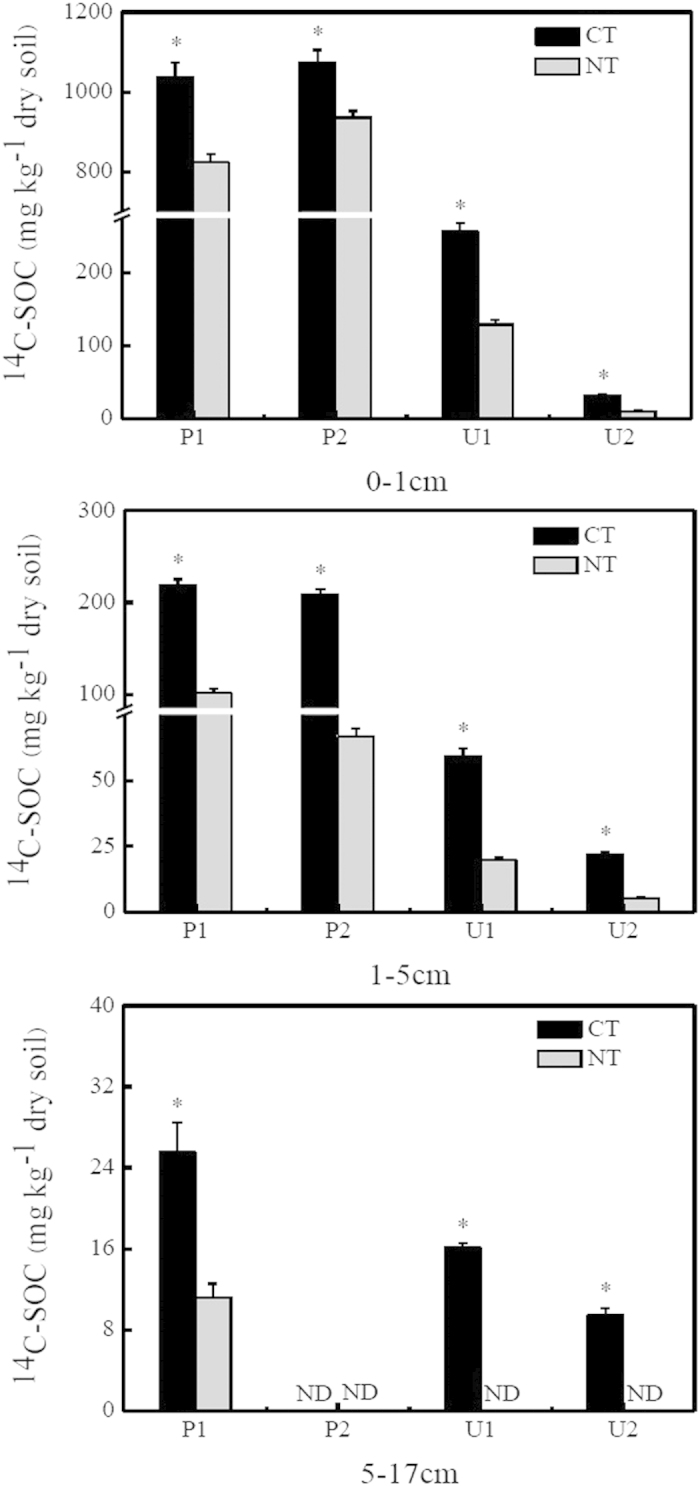
The ^14^C-SOC concentrations recovered at different depths (0–1 cm, 1–5 cm, and 5–17 cm) in conventional tillage (CT) and no-till (NT) soils after 110 days of incubation. Error bars indicate the standard error of the mean (n = 4). *indicates significant differences between CT and NT soils at *P* < 0.05; nd, not detectable.

The CT treatment significantly increased the ^14^C-SOC over the 110-day incubation period. The amount of ^14^C-SOC was, on average, 87% higher in CT soils when compared with NT soils at depths of 0–1 cm, and 210% higher at 1–5 cm (Fig. 1; Table 1). At 5–17 cm, the ^14^C-SOC concentration was 141% greater in P1 (paddy) soils under CT relative to the NT treatment, and no ^14^C-SOC content was detected under NT treatments of three other soils (Fig. 1). Generally, different types of soils responded differently to tillage treatments: CT treatment had a greater impact on upland soils than on paddy soils (Fig. 1; Table 1). Under both CT and NT treatments, the overall ^14^C-SOC concentrations decreased with increasing soil depth, with deeper soil layers being more sensitive to tillage practices (Fig. 1; Table 1). ANOVA analyses revealed no significant interactive effect of soil type, soil depth, and soil tillage on the measured ^14^C-SOC content (Table 1).

### The incorporation of ^14^C into MBC and DOC

**Figure 2:**
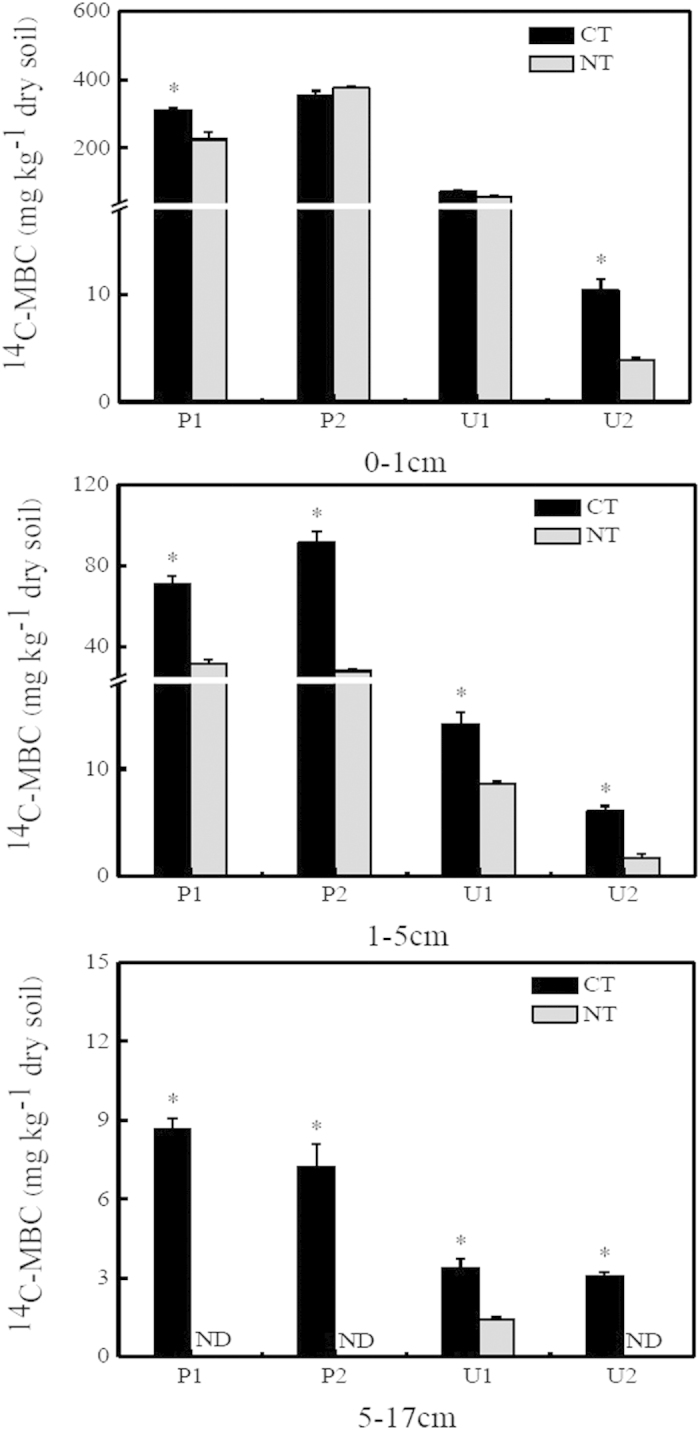
The ^14^C-MBC concentrations recovered at different depths (0–1 cm, 1–5 cm, and 5–17 cm) in conventional tillage (CT) and no-till (NT) soils after 110 days of incubation. Error bars indicate the standard error of the mean (*n* = 4). *indicates a significant differences between CT and NT soils, *P* < 0.05; nd, not detectable.

**Figure 3:**
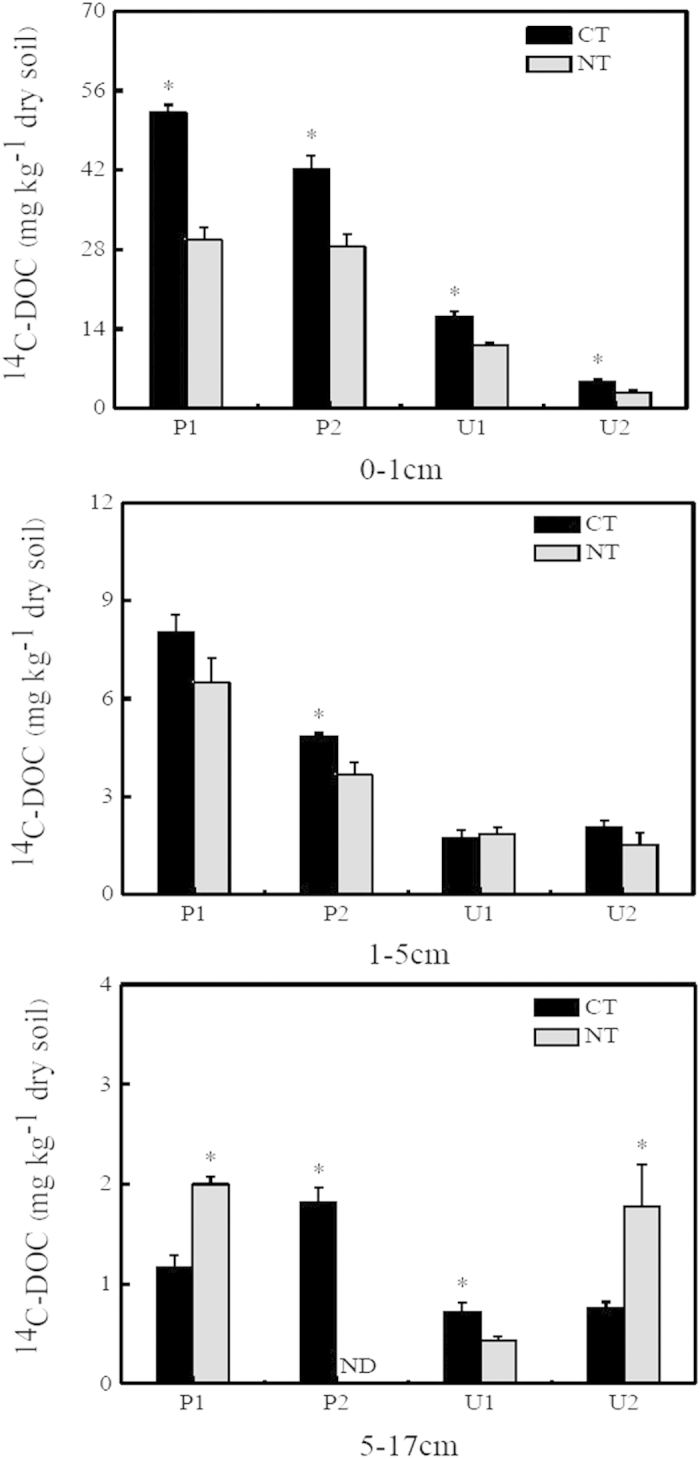
The ^14^C-DOC concentrations recovered at different depths (0–1 cm, 1–5 cm, and 5–17 cm) in conventional tillage (CT) and no-till (NT) soils after 110 days of incubation. Error bars indicate the standard error of the mean (*n* = 4). *indicates significant differences between CT and NT soils, *P* < 0.05 level; nd, not detectable.

The incorporation rates of autotrophically fixed ^14^C into microbial biomass carbon (MBC) and dissolved organic carbon (DOC) were modulated by soil tillage (Fig. 2, 3; Table 1). Larger amounts of ^14^C-MBC were recovered from CT soils than from NT soils, but the difference was not significant in P2 (0–1 cm) and U1 (upland soil; 0–1 cm) (Fig. 2; Table 1). No significant interaction between soil type, soil depth, and soil tillage was observed to affect ^14^C-MBC concentration. Compared with NT treatments, CT also significantly increased ^14^C-DOC concentration in the 0–1 cm depth by an average of 33%, whereas the effect of tillage on ^14^C-DOC contents at greater depths was highly dependent on soil type (Fig. 3; Table 1). Under both CT and NT treatments, larger amounts of ^14^C-MBC and ^14^C-DOC were observed in paddy soils when compared with upland soils at 0–1 cm and 1–5 cm (Fig. 2), with a significant soil type × soil tillage interaction (Table 1). Both ^14^C-MBC and ^14^C-DOC contents decreased with increasing soil depth in CT and NT soils, although a significant soil depth × soil tillage interaction was only observed with ^14^C-DOC (Figs 2 and 3, Table 1). Additionally, a significant interactive effect on ^14^C-DOC concentration was observed among soil type, soil depth, and soil tillage.

### Bacterial and cbbL gene abundance

The bacterial (*16 S rRNA*) and *cbbL* gene abundance differed across tillage managements, as well as across soil type and depth (Table 2). Lower bacterial and *cbbL* gene abundances were observed in CT treatments when compared with NT, but only in P1, P2, and U2 soils. In contrast, higher *cbbL* gene abundance was observed in U1 soil under CT and NT treatments (Table 2). Soil type exhibited a significant effect on bacterial and *cbbL* gene abundance, with paddy soils generally being more susceptible to tillage than upland soils (Tables 1 and 2). In both CT and NT soils, *cbbL*-carrying bacteria decreased with increasing soil depths in paddy soils, but increased with depth in upland soils, with no significant interaction being observed among soil type, soil depth, and soil tillage (Table 1).

### The specific CO_2_ fixation activity of autotrophic bacteria

The CT treatments enhanced the specific CO_2_ fixation activity compared to NT treatments, irrespective of soil type and depth, with the increase being significant in all soils except U1 (Tables 1 and 2). Significant soil type × soil tillage and soil depth × soil tillage interactions were observed, suggesting that soil tillage affects CO_2_ fixation activity differently depending on the specific soil type or depth (Tables 1 and 2).

## Discussion

We were able to clearly detect ^14^C in soils of both the CT and NT treatments, indicating that CO_2_ fixation had taken place (Fig. 1). Mechanistically, both biotic and abiotic processes could be responsible for the recovery of ^14^C labelled pools in soils. For example, Miltner *et al.*^25^ documented that after 81 days of incubation, 0.83 μmol·g^−1^ soil of ^14^CO_2_ was fixed to biologically active soil, with 96% bound as organic compounds. However, approximately 0.02 μmol·g^−1^ soil of ^14^CO_2_ was detected in the fumigated control soil, which did not have microbial activity, and 91% of this fixed ^14^C was bound as carbonates. Similar results were observed in studies with longer incubation time or different tracers (e.g. ^13^CO_2_)^26^,^27^. In this study, we were able to remove all ^14^C bound as carbonates via the concentrated H_2_SO_4_–H_3_PO_4_ treatment, allowing us to conclude that observed CO_2_ fixation was mainly from biological processes. Within these processes, we discounted heterotrophic fixation as a primary route because our previous study did not detect fixed ^14^C in dark-incubated soils^4^. Therefore, we concluded that microbial autotrophs are the primary source of carbon fixation.

Crucially, significantly higher amounts of ^14^C fixation occurred in the CT treatments compared with the NT treatments (Fig. 1). However, tillage effects on the abundances of total bacteria and *cbbL*-carrying bacteria were variable among soils. Both decreases and increases of 16 S rRNA and *cbbL* gene numbers were observed under CT treatment. Negative effects of tillage on soil microbial abundance were found in P1, P2, and U2 soils, irrespective of soil depth. These data support previous studies showing that tillage negatively affects the abundances of diverse functional groups such as denitrifiers and nitrifying microorganisms^28^,^29^,^30^. For example, tillage reduced denitrifier populations in loam soils with wheat/fallow rotation^28^, and after 22 years of conventional tillage, the abundance of ammonia-oxidising bacteria was found to decrease in a subtropical rice-based ecosystem^29^. These studies have suggested several underlying mechanisms to explain tillage effects on microbial numbers, including the disruption of soil aggregates by CT that alters nutrient availability and intensifies carbon source preemption^28^,^29^,^30^. The suppression of total bacterial and *cbbL*-carrying bacterial abundance in the P1, P2, and U2 soils of this study may have been caused by similar mechanisms.

Interestingly, we found that CT exerted the opposite effect on bacterial abundance in U1 soil (Table 2), which is a vegetable soil subject to frequent tillage, in contrast to the other three soils. Tillage frequency influences the threshold rates of aggregate turnover; when the thresholds are passed, organic carbon is actually retained in, rather than released from, tilled soil aggregates^31^. Thus, we propose that the CT treatment of U1 soil probably surpassed the threshold aggregate turnover rate, resulting in the accumulation of soil organic carbon. Additionally, we found that total P was almost twice as high in U1 soil than in the other three soils (Table 2). Phosphorus is essential to microbial growth and its availability is highly related to tillage practices^32^. Therefore, CT treatment of U1 soil may create favourable growth conditions for soil microbial communities by improving P and organic substrate availability^33^, resulting in a higher abundance of *cbbL*-containing bacteria.

The present results showed that the amount of key players in CO_2_ fixation (*cbbL*-carrying bacteria) was significantly lower for CT than NT in most soils, but microbially assimilated ^14^C was markedly higher under CT conditions than under NT, indicating that the population size of *cbbL*-containing bacteria may not necessarily reflect their functional activities under changing soil conditions. This disparity in abundance and activity may be due to the enhancement of carbon fixation under tillage management. Previous research has demonstrated that mechanical disturbance from conventional tillage can largely modify soil physical properties such as bulk density and porosity^34^,^35^,^36^,^37^. For instance, Gruber *et al.*^34^ reported that ploughed soils exhibited a lower bulk density than no-till soil (1.1 g cm^−3^ versus 1.3 g cm^−3^), and tillage also lowered total porosity compared with no-till conditions^35^. These differences in soil porosity and soil bulk density affect the contact between *cbbL*-carrying bacteria and their substrate, ^14^CO_2_^36^,^37^, improving soil gas diffusivity^37^. In turn, more ^14^CO_2_ is likely to be supplied, thus stimulating the CO_2_ assimilation activity of *cbbL*-bearing bacteria. Our data support this hypothesis because CT soils exhibit greater specific CO_2_ fixation activity than NT soils (Table 2). Previous research has also demonstrated that tillage practices enhance light transmittance^38^ and hydraulic conductivity^39^, as well as create novel ecological niches^40^. All of these factors substantially enhance the CO_2_ fixation activity of *cbbL*-bearing bacteria^4^,^8^, leading to our observation of greater fixed ^14^C under CT treatment. Moreover, we can assume that tillage management, in altering soil properties, may also affect alternative CO_2_ fixation pathways that are sensitive to such changes, thereby contributing to the differences in soil autotrophic microbial CO_2_ fixation between CT and NT treatments. We hope that future analyses will resolve these possibilities.

Generally, we observed a consistent trend across both CT and NT treatments, where the microbial fixed ^14^C concentrations are higher in paddy soils than upland soils, although this pattern was less obvious at deeper soil depths (Fig. 1, Table 1). We believe this result was caused by the anaerobic conditions in flooded paddy soils, which were flooded with 1–2 cm of sterile water during incubation. The anaerobic paddy soils, in contrast with the aerobic environment of upland soils, may have provided favourable anaerobic niches that promoted the activity of autotrophic CO_2_ fixation bacteria and slowed the decomposition of newly fixed ^14^C^8^,^22^.

In this study, fixed ^14^C amounts also varied across different soil depths for both CT and NT treatments (Fig. 1, Table 1). Specifically, conventional tillage had a more pronounced effect on deeper soils than on topsoil, increasing the differences in CO_2_ fixation rate at 1–5 cm and 5–17 cm compared with 0–1 cm. Previous studies have shown that photoautotrophs are the main contributors to surface soil CO_2_ fixation, whereas chemoautotrophs may be involved in CO_2_ assimilation in deeper soil layers by using inorganic compounds as electron donors^23^. Without tillage, soils are more compact and less porous, conditions that will slow ^14^CO_2_ and H_2_ diffusivity, as well as inorganic substrate transfer, down the soil profile. Relative to tilled soil, this inhibition is more pronounced at deeper depths for un-tilled soil^41^. Because ^14^CO_2_, H_2_, and inorganic compounds are important electron donors for chemoautotrophic bacterial CO_2_ fixation, variation in the vertical stratification of electron donors down the soil profile across CT and NT treatments will likely exert differential effects on chemoautotrophic bacterial activity^23^. Moreover, our previous study indicated that a portion of the fixed ^14^C in deep soil layers probably originated from the downward translocation of microbially assimilated C at the soil surface^23^. Tillage-induced changes in soil structure, such as increases in bulk density and decreases in porosity, may therefore benefit the transfer of microbially assimilated C down the soil profile, providing an explanation for the differential impact of tillage at varying soil depths.

## Methods

### Soil sampling

The experiment was carried out with two paddy soils (P1, P2) and two upland soils (U1, U2) from different regions of Hunan Province, in the subtropical region of China. The properties of the soils before incubation are shown in Table 3. These soils cover the typical land use types in this area: P1 and P2 are from double-rice plantations, U1 is a vegetable plantation base, and U2 receives upland-crop rotation. Soil sampling was conducted in November 2010 after the final harvest of crops. For each site, two sets of soil samples, termed CT treatment soil and NT treatment soil, were prepared.

Intact soil cores (i.e. NT treatment soils) were collected directly by inserting four polyvinyl chloride (PVC) containers (10 cm diameter, 20 cm height) approximately 17 cm into soils. The PVC pipes were immediately sealed with a fitted end cap after being extracted from the sampling site and transferred to the laboratory.

To establish the CT treatment soil at the corresponding site, soil cores were randomly sampled using a stainless steel auger and homogenised with mixing. After plant residues and stones were removed, the mixed soil was air-dried and then sieved through a < 5 mm mesh. Before commencing the ^14^C-CO_2_ labelling experiment, the air-dried soils were rewetted using distilled water (P1 and P2, flooding; U1 and U2, 45% water holding capacity [WHC]), and all soils were equilibrated for 2 weeks to stabilise microbial activity. Finally, four CT treatment soils were obtained by packing the pre-incubated sieved soils into PVC containers (10 cm diameter, 20 cm height) to a depth of approximately 17 cm, equivalent to the depth of the NT treatment soil. Four replicates of each soil type (P1, P2, U1, U2) were prepared for each treatment.

### Incubation experiment design

Microcosms of soils under CT and NT treatments were placed into an airtight growth chamber (80 × 250 × 120 cm). Soils were incubated for 110 days with ^14^C-CO_2_ produced by the reaction between Na_2_^14^CO_3_ (at a radioactivity of 1.65 × 10^4^ Bq·mL^−1^) and HCl (2 M). The concentration of ^14^C-CO_2_ in the incubation system was maintained at about 350 μL·L^−1^. The concentration of CO_2_ in the growth chamber was monitored with an infrared CO_2_ sensor (GasCard NG, 6132 A, Guangzhou, China). During the incubation period, lamps generating artificial light (intensity: 500 mmol photons m^−2^·s^−1^ PAR) were open from 08:00 am to 08:00 pm each day. Day/night temperatures were set at 31 ± 1 °C/24 ± 1 °C, respectively, and relative atmospheric humidity was held at 80–90%. Paddy soils were incubated by flooding with a 1–2 cm water layer while upland soils were kept drained (45% WHC) during the incubation period. Soil moisture was also determined at harvest and was nearly identical to the soil water content at the beginning of the experiment (data not shown). At the end of the incubation period, each soil column in the PVC container was sectioned from the top into three intervals (0–1 cm, 1–5 cm, and 5–17 cm). The sectioned soils were stored separately in three parts. One part was dried for the determination of ^14^C-SOC content and another portion was immediately used to measure ^14^C-MBC. The remaining part was stored at −70 °C for molecular ecological analysis. Soil moisture content was measured by oven-drying the soil at 105 °C immediately after sampling.

### Soil property analysis

Soils were air-dried and sieved (2 mm) for SOC and total nitrogen measurements, which were performed with dry combustion using a macro elemental analyser (Vario MAX C/N, Elementar, Germany). Total phosphorus was measured using the Mo-Sb colorimeteric method^42^. Soil pH was measured in suspension using a soil:H_2_O ratio of 1:2.5 (w/v). Soil clay content was determined using the pipette method^43^, and cation exchange capacity was measured according to the procedure detailed by Thomas^44^.

### Soil ^14^C radioactivity analysis

To remove inorganic carbon (such as CaCO_3_) from soil samples, 3.0 mL 2.5 M HCl was added and mixed with 1.50 g of soil (sieved with a mesh <0.149 mm) (v:w = 2:1) in Dolphin tubes for 24 hours. Then, prior to measuring ^14^C-SOC, aliquots were washed twice with 3.0 mL H_2_O to remove any remaining HCl. Post-washing, 1.50 g of the concentrated, HCl-treated dried soil was transferred to a flask containing K_2_Cr_2_O_7_ (0.2 M, 20 mL) and concentrated H_2_SO_4_–H_3_PO_4_ (v:w = 5:1). This mixture was digested at 165 °C for 8 min under continually replenished pure O_2_ and for 10 min without O_2_ thereafter^22^. The evolved CO_2_ was trapped with NaOH (0.4 M, 40 mL), and the ^14^C radioactivity was measured using an automated liquid scintillation counter (LS-6500, Beckman, Germany). The measurement of ^14^C-MBC was performed with the fumigation-extraction method, and the amount of ^14^C-DOC was determined using K_2_SO_4_ extracts of non-fumigated soil^38^. Finally, ^14^C-SOC, ^14^C-MBC, and ^14^C-DOC concentrations were calculated according to the procedure described by Ge *et al.*^22^, with additional details available in our previous reports^4^,^22^,^23^.

### Soil DNA extraction

The extraction of soil microbial DNA was performed in triplicate using a FastDNA Spin Kit, following manufacturer protocol (BIO101, Qbiogene Inc., Carlsbad, CA). The DNA extracts were resuspended in sterilised water for quality and quantity checks. The amount of extracted DNA was determined with a spectrophotometer (Nanodrop ND-1000, PeqLab, Germany), and the quality was evaluated using 1% agarose gel electrophoresis.

### Bacterial (16 S rRNA) and cbbL gene abundance analysis

Quantification of the bacterial (*16* *S rRNA*) gene and the *cbbL* gene was performed with real-time quantitative PCR, using the primers described by Yuan *et al.*^8^ and Wu *et al.*^23^ respectively. Gene copy numbers were quantified in triplicate using the primers 799 F (5′-ACCMGGATTAGATACCCKG-3′) and 1492 R (5′-ACGGTTACCTTG- TTACGACTT-3′)^8^ for *16* *S rRNA*, as well as the primers K2f (5′-ACCAYCAAG CCSAAGCTSGG-3′) and V2r (5′-GCCTTCSAGCTTGCCSACCRC-3′)^24^ for *cbbL*. The quantification followed previously described procedures^23^ using a *cbbL* cloned standard for constructing standard curves. Briefly, the target *cbbL* gene fragment was amplified from extracted DNA and the generated PCR amplicons ligated into the pGEM-T Easy Vector. The vectors were then transformed into *Escherichia coli* DH5α competent cells following manufacturer protocol (Promega, Mannheim, Germany). Randomly chosen white colonies were sequenced, and positive clones with target inserts were used for plasmid DNA extraction. Ten-fold serial dilutions of plasmid DNA were subjected to quantitative PCR in triplicate to establish the standard curve. Negative controls without template DNA were run in parallel with template DNA for the soil *cbbL* genes. The reaction was performed in 10 μL reaction mixtures containing: 5 ng template DNA, 5 μL SYBR Premix Extaq (Takara Bio Inc., Shiga, Japan), and 0.1 μM of each primer, following previously described thermal cycling conditions^23^. The copy numbers of the *16* *S rRNA* and bacterial *cbbL* gene in the reaction mixture were automatically calculated using SDS 2.3 software within the Real-Time PCR System, with reference to the standard curve generated for each run.

### Specific CO_2_ fixation activity

The CO_2_ fixation activity per *cbbL* copy was defined as the specific CO_2_ fixation activity of the autotrophic bacteria and was calculated by dividing the observed ^14^C-SOC concentration by the detected *cbbL* gene copy number.

### Statistical analysis

All data are expressed as means with standard errors. Differences in ^14^C radioactivity and *cbbL* gene abundance between CT and NT soils were tested using independent samples *t* tests. The effects of soil type, soil depth, soil disturbance, and their interactions on autotrophic bacterial CO_2_ uptake rate, bacterial abundance, and specific CO_2_ fixation activity were evaluated using univariate ANOVA. Significance for all tests was set at *P* < 0.05, and analyses were performed in SPSS 13.0 for Windows (IBM, Armonk, NY, USA).

## Additional Information

**How to cite this article**: Ge, T. *et al.* Effect of simulated tillage on microbial autotrophic CO_2_ fixation in paddy and upland soils. *Sci. Rep.* **6**, 19784; doi: 10.1038/srep19784 (2016).

## Figures and Tables

**Table 1:** Results of ANOVA investigating the effects of soil type, depth, tillage, and their interactions on ^14^C-SOC concentration, ^14^C-MBC concentration, ^14^C-DOC concentration, *cbbL* gene copies, and specific CO_2_ fixation activity of autotrophic bacteria.

Factors	^14^C-SOC (mg·kg^−1^ dry soil)		^14^C-MBC (mg·kg^−1^ dry soil)		^14^C-DOC (mg·kg^−1^ dry soil)		*cbbL* gene copies (10^9^copies g^−1^ dry soil)		Specific CO_2_ fixation activity (10^−7^ g per copy)	
	*F*	*P*	*F*	*P*	*F*	*P*	*F*	*P*	*F*	*P*
Soil type	421.27	<0.001	112.54	<0.001	1385.00	<0.001	64.09	<0.001	19.25	<0.001
Soil depth	450.65	<0.001	113.57	<0.001	359.68	<0.001	4.43	0.017	6.59	0.013
Soil tillage	47.6	<0.001	8.93	0.005	103.04	<0.001	67.65	<0.001	18.26	<0.001
Soil type × Soil depth	284.3	<0.001	67.72	<0.001	229.25	<0.001	6.23	0.004	22.48	<0.001
Soil type × Soil tillage	11.12	0.002	4.79	0.034	21.97	<0.001	54.98	<0.001	8.11	0.001
Soil depth × Soil tillage	5.06	0.029	0.11	0.742	82.30	<0.001	4.58	0.038	5.33	0.007
Soil type × Soil depth × Soil tillage	0.07	0.795	0	0.980	22.14	<0.001	4.00	0.052	0.02	0.883

DOC, dissolved organic carbon; MBC, microbial biomass carbon; SOC, soil organic carbon.

*F* and *P* values are results of the ANOVA, with the following factors: soil type (paddy soil, upland soil), soil depth (0–1 cm, 1–5 cm, and 5–17 cm), and soil tillage (CT soil, NT soil).

**Table 2:** The abundance and specific CO_2_ fixation activity of autotrophic bacteria at different depths of CT and NT soils.

Soil depth	Soil type	Bacterial (*16S rRNA*) Abundance (10^11^ copies g^−1^ dry soil)		*cbbL* Abundance (10^9^ copies g^−1^ dry soil)		Specific CO_2_ fixation activity (10^−7^ g per copy)	
		CT soil	NT soil	CT soil	NT soil	CT soil	NT soil
0–1 cm	P1	0.86 ± 0.01 b	1.58 ± 0.01 a	0.58 ± 0.05 b	1.65 ± 0.08 a	18.10 ± 1.49 a	5.04 ± 0.33 b
	P2	0.59 ± 0.06 b	1.86 ± 0.02 a	0.38 ± 0.11 b	2.98 ± 0.50 a	31.25 ± 6.01 a	3.33 ± 0.44 b
	U1	0.41 ± 0.05 a	0.27 ± 0.03 b	0.26 ± 0.06 a	0.13 ± 0.01 a	10.85 ± 1.82 a	10.10 ± 1.17 a
	U2	0.14 ± 0.02 b	0.39 ± 0.04 a	0.03 ± 0.01b	0.30 ± 0.13 a	10.40 ± 1.75 a	0.42 ± 0.00 b
1–5 cm	P1	0.73 ± 0.05 b	1.28 ± 0.07a	0.36 ± 0.01 b	1.11 ± 0.23 a	6.09 ± 0.16 a	0.94 ± 0.02 b
	P2	0.36 ± 0.04 b	1.27 ± 0.13 a	0.07 ± 0.01 b	1.16 ± 0.19 a	29.36 ± 2.84 a	0.57 ± 0.00 b
	U1	0.37 ± 0.04 a	0.30 ± 0.01a	0.33 ± 0.04 a	0.13 ± 0.01 b	1.87 ± 0.33 a	1.49 ± 0.00 a
	U2	0.12 ± 0.01 b	0.29 ± 0.02 a	0.16 ± 0.06 a	0.39 ± 0.14 a	1.81 ± 0.41 a	0.02 ± 0.00 b
5–7 cm	P1	0.74 ± 0.06 b	1.08 ± 0.07 a	0.34 ± 0.06 b	0.99 ± 0.08 a	1.24 ± 0.44 a	0.01 ± 0.00 b
	P2	0.32 ± 0.04 b	0.86 ± 0.06 a	0.06 ± 0.01 b	0.66 ± 0.04 a	−	−
	U1	0.38 ± 0.04 a	0.31 ± 0.04 a	0.52 ± 0.08 a	0.13 ± 0.00 b	0.30 ± 0.00 a	−
	U2	0.11 ± 0.01 b	0.27 ± 0.02 a	0.16 ± 0.03 b	0.34 ± 0.04 a	0.51 ± 0.00 a	−

Different lower case letters represent significant differences (P < 0.05) in CT and NT soils.

**Table 3:** Basic study site information and corresponding soil physicochemical characteristics.

Soil	Location	Land use	pH	SOC (g·kg^−1^)	Total N (g·kg^−1^)	Total P (g·kg^−1^)	Clay content (%)	CEC (cmol·kg^−1^)
P1	Ganshan	Double-rice	6.15	21.89	2.64	1.05	39.76	11.79
P2	Pantang	Double-rice	5.66	20.93	2.81	0.70	33.19	13.16
U1	Huangxing	Vegetable	6.60	18.73	2.57	3.65	26.12	13.69
U2	Pantang	Rape-corn	4.40	6.19	1.39	0.75	31.38	11.05

CEC, cation exchange capacity; SOC, soil organic carbon.
